# Aftermath of the COVID-19 Pandemic on Mental Health and Well-Being of Patients With Thalassemia Major in Pakistan: A Qualitative Study

**DOI:** 10.7759/cureus.35048

**Published:** 2023-02-16

**Authors:** Muhammad Hammad, Rasikh Arif, Sehar Bano, Usman Ghani, Hima Bindu Reddy Basani, Vivek Sanker

**Affiliations:** 1 Pharmacy, Shifa Tameer-E-Millat University Shifa College of Pharmaceutical Sciences, Islamabad, PAK; 2 Clinical Research, Al-Shifa Trust Eye Hospital, Rawalpindi, PAK; 3 Internal Medicine, Riphah School of Leadership, Riphah International University, Rawalpindi, PAK; 4 Clinical Research, Clinical Trials Unit, Aga Khan University Hospital, Karachi, PAK; 5 Graduate Medical Education, Lyceum Northwestern University, Dagupan, PHL; 6 General Surgery, Noorul Islam Institute of Medical Sciences and Research Foundation, Trivandrum, IND

**Keywords:** well-being, thalassemia major, pakistan, mental health, covid-19

## Abstract

Background and aim

Chronic patients with thalassemia major were mainly recognized as more prone to poor mental health during this global pandemic. This study aims to evaluate causal relationships leading to poor outcomes and how they manage to tackle this.

Methods

In-depth face-to-face semi-structured interviews were conducted with 21 thalassemia patients selected through probability consecutive sampling from Pakistan Thalassemia Welfare Society Centers. The following criteria served as the basis for the interview: (1) solitude at home, (2) interruption of transfusion services, (3) alteration of appetite and interests, (4) lack of control and uncertainty, (5) extensive media coverage, (6) deterioration of pre-existing health issues, difficulty in sleeping, (7) practicing gratitude, (8) participation in activities and hobbies, (9) connectivity with others, and (10) ability to recognize social support. All interviews were recorded, transcribed, and analyzed with reflexive thematic analysis.

Results

The commonly described mental health conditions were depression and concern about the overall health status. The following seven elements associated with the pandemic contributed to the deterioration of mental health: (1) isolation at home, (2) disruption in transfusion services, (3) change in appetite and interests, (4) lack of control and uncertainty, (5) intensive media reporting, (6) worsening of pre-existing health problems, and (7) difficulty in sleeping. The following four coping strategies were identified for maintaining mental issues: (1) practicing gratitude, (2) involvement in activities and hobbies, (3) connectivity with others, and (4) discerning social support.

Conclusions

Thalassemia major patients had been negatively affected during the pandemic. Only a small number of people modified their management techniques to maintain steady well-being.

## Introduction

Thalassemia is a chronic and inherited disease in which there is a reduced tendency of the body to produce adequate amounts of hemoglobin. This disease has a high occurrence in South East Asia, South Asia, Middle East, and Mediterranean countries [1]. WHO states that “hemoglobin disorders have affected 71% of countries and 50,000 to 100,000 children die each year with thalassemia major in developing countries.” Thalassemia affects not only the physical health of patients but also their mental health. It directly impacts the well-being and mental health of the affected person. Thalassemia patients require regular blood transfusions throughout their life. They also need daily treatment of chelation to eliminate the excess iron from their blood. Excess iron can cause organ damage and ultimately organ failure [2]. In addition, periodic screening of complications and psychological counselling are also required. It is associated with other comorbidities including pulmonary hypertension, diabetes, and other cardiac problems.

In December 2019, in the city of Wuhan, China, severe acute respiratory syndrome (SARS) erupted, which is known as the coronavirus disease 19 (COVID-19). This pandemic affected the entire world and badly affected the daily life of every individual [3]. To control the deadly virus, lockdown was administered countrywide, thus restricting the activities of daily life. Hospitals were only focused on COVID-19 and trying to cope with this disease. Thalassemia patients found it very difficult to go to the clinics for regular blood transfusions. Moreover, there was a reduction in the number of voluntary blood donations, thus causing more hurdles and difficulties for thalassemia patients. Due to the lockdown, access to the hospital and clinics was reduced, posing great threat to the psychiatric and physical well-being of thalassemia victims. This lifetime treatment and emergency of COVID-19 exacerbated the depression, anxiety, and mental disorders among thalassemia patients.

There is a great association between mental health disorders and thalassemia patients, and COVID-19 pandemic has added fuel to the fire. To be healthy and to cope with the burden of disease, mental health is very crucial. COVID-19 has affected both the physical and mental health of people. Quarantine and lockdown caused fear among masses and affected the thalassemia patients as well. These patients are more prone to be affected with mental health disorders and are subject to special care and attention. The purpose of this research is to deeply analyze the condition of thalassemia patients in the COVID-19 era and its association with mental stability and well-being of the patients, along with the strategies adopted by the patients to cope with the ongoing situation. This area is still needed to be explored as little work is done in this domain. With the help of this research, we can find out the problems of thalassemia patients and identify the strategies to overcome those problems related to mental health and well-being of patients [2].

A study conducted by Alsaad in 2020 showed that there is an upraised ratio of psychiatric disorders, specifically depression and anxiety, in thalassemia patients. Among all, depression is the most common psychological mental disorder [4]. Depression and thalassemia have bi-directional relationships in these patients. Depressed children had the highest rate of fatigue, pain, discomfort, and more sleep disturbance. Nasiri et al. assessed that thalassemia patients are at a higher risk of psychiatric disorders as compared to the general population and are subject to further consideration [5]. Further observations and consultations are needed, and diagnostic tests are recommended to achieve deeper and confident data. A 2009 research study by Azarkeivan et al. on the associates of poor physical and mental health related quality of life found that depression is linked to both poor physical and mental health in patients of major and intermedia and that somatic comorbidities and anxiety are associated with poor physical and mental health, respectively [6]. In a research study in 2012, Naderi et al. studied the assessment of mental stability and associated factors among thalassemia major patients in South East of Iran and concluded that there is a high prevalence of psychological disorders among thalassemia patients especially girls. In this study, depressive disorders had the highest prevalence followed by anxiety disorders, somatic disorder, and social dysfunction [7]. In 2021, Cerami et al. conducted a cross-sectional study on how unpredictable crisis span affects psychosocial dimensions in beta-thalassemia patients during the COVID-19 pandemic and concluded that patients had higher anxiety levels and a predominant transcendent coping profile to overcome social isolation and habit change in COVID-19, which are particularly damaging in insubstantial thalassemia patients [8].

## Materials and methods

A qualitative descriptive study was conducted using a convenient sample of 21 volunteer subjects (13 males and 8 females) with thalassemia major, with a mean age of 14.1 years (SD = 6.5 years). The initial data of the patients were taken from Pakistan Thalassemia Welfare Society Centers. In-depth face-to-face semi-structured interviews were conducted to illustrate the impact of living with thalassemia major during the era of COVID-19 crisis.

Ethical approval of the study was taken from the School of Public Health, Al-Shifa Trust Eye Hospital, Rawalpindi, Pakistan (Reference No. ERC-63/AST-21). The questionnaire designed was simple, clear, and convenient enough to assess psychiatric health and well-being of thalassemia victims and to identify the coping strategies to overcome the effects. Table 1 shows the interview guiding questions for themes on deterioration of mental health.Table 2 shows the interview guiding questions used to identify coping strategies.

**Table 1:** Interview guide focusing on themes for coping strategies

Practicing gratitude	1. How are you coping with the COVID? 2. Do you practise gratitude? If yes, how?
Involvement in hobbies and other activities	1. How do you spend your day during the lockdown? 2. Your best way to spend time during lockdown?
Staying connected with others	1. How do you stay connected with others? (explain)
Discerning social support	1. How does the thalassemia center play its role? 2. Did you get support from your family members and friends?

**Table 2:** Interview guide focusing on themes for deterioration of mental health

S. no.	Theme	Questions
1.	Isolation	How acceptable is it for you to be isolated at your place? Since how many days haven’t you met anyone? How can you relate isolation and deterioration of mental health to COVID-19?
2.	Blood transfusion services	How often do you go for blood transfusion? How did the lockdown affect your visit to the hospital for blood transfusion? So how do you relate the effect of isolation and transfusion service interruption on your mental health?
3.	Change in appetite and interests	Did COVID-19 impact your appetite? How often did you eat before the pandemic? (number of meals a day) How often do you eat now? What do you like to have in your meal? Favorite meal/food? How often did you have it before the pandemic and now? Do you find any difference in your appetite before and during a pandemic? Tell me about your hobbies and interests. Has it forced you to change your interests? If so, how do you explain it?
4.	Lack of control and anxiety	How often do you feel sad/anxious? How do you define sadness/happiness/anxiety/depression? How do you relate to your (sadness/happiness/anxiety/depression) before and during the pandemic?
5.	Worsening of overall health	How do you feel about your health? Rate your health condition (1 to 10). Did your health deteriorate during COVID? How? Explain.
6.	Disruption in sleep pattern	How many hours in a night/day do you sleep (before and during the pandemic)? Explain the impact of COVID on your sleep pattern. How do you relate the depression to your health condition, media reporting, sleep pattern, etc.

The designed questionnaire was developed for this study using existing theories on health and social networks [9], behavior change [10], and stress and coping [11]. All interviews were recorded, coded, transcribed, and analyzed with reflexive thematic analysis following the steps outlined by Braun and Clarke [12]. Themes of mental health and well-being status alone with coping strategies are as follows: isolation at home, disruption in transfusion services, change in appetite and interests, lack of control and uncertainty, intensive media reporting, worsening of pre-existing health problems, difficulty in sleeping, practicing gratitude, involvement in activities and hobbies, connectivity with others, and discerning social support.

## Results

A total of 21 people with thalassemia major took part in the study, the majority of individuals were males (n=13, 61.9%), and the mean age of volunteers was 14.1 years (SD = 6.5 years). Summary of themes and subthemes present in Table 3 and Table 4.

**Table 3:** Frequency (%) of themes relating to deterioration of mental health

S. no.	Themes	Percentage (%)
Q1	Isolated at home	91
Q2	Disruption in transfusion services	73
Q3	Change in appetite and interests	58
Q4	Lack of control and anxiety	89
Q5	Intensive media reporting (anxiety, depression)	96
Q6	Worsening of overall health	83
Q7	Sleep pattern disruption	69

**Table 4:** Frequency (%) of themes relating to coping strategies

S. no.	Themes	Percentage (%)
Q1	Practicing gratitude	69
Q2	Involvement in hobbies and other activities	87
Q3	Staying connected with others	55
Q4	Discerning social support	48

Themes relating to the deterioration of mental health

Theme 1: Isolation at Home

Out of 21 participants, 18 (96%) described isolation at home as one of the most common deteriorating factors of mental health. Most of the participants stated that staying at home for long hours is disrupting their routine as well as their interpersonal relationships in their lives. This theme includes acceptance of patients of the situation, the number of days they were isolated at home, and the impact COVID-19 and isolation had on them. All the patients described the pandemic as a stressful situation as it had the potential to take a toll on their physical as well as mental health.

“I thought the situation would get better with time but it didn't. lack of normal human interaction as usual like meeting my friends made me feel lonely and tired throughout the day, I am still trying to adapt to the pandemic and accept the situation.” (Patient 2)

“I have been isolated for about two weeks now. I worry a lot about the well-being of those who live far away from me. Not being able to see my family who has been affected by COVID-19 had a lot of impact on me.” (Patient 7)

 “Being an introvert I don't feel the effects of isolation very early on but at a times I was really worried if I would ever get any help if I fell sick.” (Patient 13)

Theme 2: Disruption in Transfusion Services

Insufficient storage of the blood,unavailability of blood donation services, and unavailability of public transportation resulted in disruption in transfusion services, leading to deterioration of mental health in around 73% of the patients.

“I used to go for a blood transfusion once every two weeks, but due to lack of proper public transportation, someone else had to drive me to the hospital. So, I missed the transfusion sessions.” (Patient 9)

“We needed permission letters to travel to the hospital during complete lockdown; this lack of extra paperwork had led to few problems during travelling to the hospital and I worried a lot about my health.” (Patient 18)

Theme 3: Change in Appetite and Interests

Disruption in lifestyle and a decrease in quality of life have contributed to the deterioration of mental health in 58% of the patients.

 “I used to have at least 3 meals a day before pandemic, but due to lack of physical activity and minimal interaction with people, my appetite has gone down and I also started losing weight” (Patient 15)

“My appetite has gone down drastically. I eat around one to two meals a day and sometimes even in the middle of the night, which has caused me a lot of health problems.” (Patient 16)

“I used to go for a walk every evening before the pandemic but now I stay at home and try to watch television or listen to some music.” (Patient 20)

“I have lost interest in my hobbies like reading books. I don't get to visit my grandchildren and children like before, which has led to loss of interest in my daily interests.” (Patient 17)

Theme 4: Lack of Control and Anxiety

Around 89% of the participants reported that the pandemic has led to disruption in various areas of life causing them a feeling of lack of control over situations, lifestyle, and deterioration of mental health and well-being.

“Waking up in the morning was very hard during the pandemic; the number of deaths due to COVID-19 had added to my anxiety. I haven't felt anxious at this frequency before.” (Patient 12)

“It was months since I saw my close family and friends. This made me lonely and sad a lot of times during the pandemic.” (Patient 18)

Theme 5: Intensive Media Reporting

This contributed to the highest (96%) level of impact on patients’ mental health. Patients reported having experienced feelings of anxiety and depression due to intense and extensive media coverage during the pandemic.

“I was a nurse by profession, and the misinformation that was spread through media made me worry a lot about mental health effects of COVID-19 on other people who aren't a part of healthcare.” (Patient 16)

 “The continuous reporting on television and seeing the lack of facilities in hospitals for patients with COVID-19 and the high rate of deaths had made me anxious a lot of times.” (Patient 20)

Overall health: All the factors mentioned above indirectly contributed to the disruption of the regular lifestyle of the patient, thereby having an overall impact on the patient's health which was reported by 83% of the patients.

“I don't feel great about my health due to disruptions in my daily lifestyle. I would rate my health as 7/10. Due to the pandemic, I don't exercise anymore, which has led to negative effects on my health.” (Patient 8)

“I don't feel healthy and active like before. It has been difficult to carry out my daily activities. I would rate my health 4/10.” (Patient 11)

Theme 6: Difficulty in Sleeping

About (69%) of the patients reported disturbances in the sleep cycle, as well as difficulty in falling asleep.

“I sleep for about five hours now. I used to sleep for about eight hours before the pandemic. I have great difficulty in falling asleep on most of the days.” (Patient 15)

“I wake up multiple times during the night. I have a broken sleep cycle now, which is also leading me to have low energy levels during the day.” (Patient 12)

Themes relating to coping strategies

The themes relating to coping strategies to maintain mental health were as follows: (1) practicing gratitude, (2) involvement in hobbies and activities, (3) staying connected with others, and (4) discerning social support. Around 87% of them were engaged in practicing hobbies and other activities to cope with the lasting effect of a pandemic.

Theme 1: Practicing Gratitude

Around 69% of participants reported practicing gratitude as a method of dealing with COVID-19.

“By starting a gratitude journal and filling it daily made me feel less anxious about the pandemic and sleep well at night.” (Patient 3)

“I expressed gratitude through daily prayers; this kept my mental health stable during these times.” (Patient 4)

Theme 2: Involvement in Hobbies and Other Activity

More than 87% of the participants used this as a coping mechanism.

“By starting daily yoga, I used to keep my body and mental health fresh and it helped me feel less anxious.” (Patient 5)

“I used to try new recipes everyday; this helped keep me busy and my mind occupied.” (Patient 2)

Theme 3: Stay Connected With Others

Around 55% of people used staying connected with others as a coping mechanism.

“I used to video call my parents every day and talk to them about what I did throughout the day and other work-related stuff.” (Patient 17)

“I and my friends had a group chat on Instagram where we would share funny posts daily and talk to each other.” (Patient 12)

Theme 4: Discerning Social Support

Around 48% looked for social support as a means of coping mechanism.

“During COVID-19, I joined many groups on Facebook where people shared their COVID-19 experiences and it felt good to know about others going through similar experiences and that you were not alone.” (Patient 15)

“I found support mainly from my family who would always give me words of encouragement whenever I felt down.” (Patient 9)

## Discussion

This is the first time, to our knowledge, that any study was conducted in Pakistan on thalassemia major patients to assess their stability physically and mentally. This study highlighted a range of themes that were specific to the mental health and well-being along with coping strategies of people living with thalassemia major [13].

Themes included anxiety and depression created by intensive media coverage of COVID-19. According to a study, the anxiety in the general population was noted to be 6.33-35.1%, with patients focusing on family members getting infected with COVID-19, while in patients with thalassemia, the levels of anxiety were noted to be higher [14]. The impact of isolation at home, disruption in transfusion services, change in appetite and interests, impact on sleep patterns, impact on overall health, and quality of life created uncertainty about the future. People having thalassemia are more impacted by ongoing situations and mental well-being along with their physical health. The results related to the findings and their implications are discussed below.

According to our study, the factors that exacerbated the factors related to the deterioration of mental health are home isolation, disruption in transfusion services, change in lifestyle, quality of life, feelings of anxiety, and overall health and sleep patterns. Hossain in 2020 in Bangladesh found that there is a disruption in blood supply because recruiting volunteers for blood donations is a big challenge in this pandemic. Moreover, samples of blood become unserviceable owing to insufficient storage, underutilization, and the short shelf-life of blood. Our results are compliant with his research in that there are fewer blood donations, thus causing difficulties for thalassemia patients and disruption in transfusion services [15].

Our findings are also similar to the study conducted by Maheri et al. in 2016, who explored the association of health-promoting healthy lifestyle among adults with beta-thalassemia major and found that both are not at an acceptable level among thalassemia patients, thus lifestyle change, uncertainty, and lack of control (quality of life) are not up to the mark, thus increasing deterioration of mental health and well-being in patients [16]. These findings are also comparable with Gollo et al.'s study conducted in 2013, which states that challenges related to the implementation of new interventions for screening and evaluation of the quality of life among thalassemia patients are more vital [17].

In 2019, Gunez conducted a case-control study regarding disturbed sleep patterns in children and adolescents with beta-thalassemia major and concluded that sleep problems are more prevalent in children and adolescents and can lead to behavioral disorders, sleep disturbance, and anxiety [18]. The results are in accordance with our results where there is a sleep problem in children with thalassemia major [12]. As per Kar et al. in 2021, people adopted several managing skills during the COVID-19 pandemic. It includes remaining busy, sharing feelings with others, talking to others, hoping for the best, and many others. This study evaluated that people adopted different strategies to cope with the anxiety and depression related to COVID-19. Patients with thalassemia in our study also tried to cope by showing gratitude, finding social support, and engaging in their activities. These results are compliant with our study [19].

According to the research by Hashemi et al. in 2015, a randomized control trial including 87 adolescents with thalassemia major concluded that teaching coping strategies to patients and self-care programs are advantageous as they can manage their anxiety, stress, and depression in a more effective manner. Problem-focused and emotion-focused strategies were intervened so that patients with thalassemia can adopt different ways to cope with the stress [20]. This research also keeps up with our results that patients adopted several strategies from their environment, applied them to their lives during the COVID-19 pandemic, and coped with their stress with different strategies.

In new research from Ahmadi in 2020, a qualitative study among 18 patients, in-depth interviews were conducted with thalassemia major patients. They explored coping strategies in terms of spiritual coping, psychosocial coping, and knowledge acquisition. Spiritual coping includes prayers, hope, and acceptance of providence. This shows that practicing gratitude is also compliant with this. Psychosocial coping was another aspect in which patients tried to cope by engaging themselves in things they love to do such as sports, listening to music, thinking positively, and comparing themselves with those who had more severe problems. They also found that support from family and society played an important part in coping with stress. This also supports the fact that engaging in hobbies, discerning social support, and being connected with others help in reducing stress and anxiety among thalassemia patients [21].

Limitations of the study

In this qualitative study, the cause-and-effect relationship cannot be established. The results cannot be generalized to all thalassemia patients as the focus group consisted of only 21 patients. Convenient sampling was done due to COVID-19, being restricted to the Pakistan Thalassemia Welfare Society Centers. A cross-national study including various institutes would yield more reliable results. There is a probability of researcher bias in these types of qualitative studies. The difficulty in identifying patterns and trends is another drawback of qualitative studies. It is always recommended to do follow-up interviews and a full-scope investigation employing statistical measures and large samples. Previous studies conducted on the neurological impact of COVID-19 on patients with mental illness can also shed light on the population of our study, and future studies can be conducted focusing on patients with thalassemia [22]. Effects of COVID-19 vaccine hesitancy and its relationship with mental health illness have been studied previously [23]. Future studies can also focus on these trends related to patients with thalassemia or any blood disorders.

## Conclusions

Data suggest that, in accordance with previous beliefs, living with thalassemia major during the COVID-19 pandemic has had detrimental effects on the health of individuals, both physically and psychologically. Depression was a common symptom, and engaging in various daily activities was the most common coping strategy employed by many patients. This research highlights the correlation between physical and mental health by age and emphasizes the importance of stress management and help-seeking. Additionally, thalassemia major patients have extended periods of isolation as a precautionary measure. This is essential in order to ensure that they are sufficiently prepared. We recommend regular screening of all thalassemia major patients. Such screening can detect areas where intervention is required in order to preserve the stability of patients during this global pandemic.
